# Clinical Outcomes of Atrial Septal Defect and Patent Foramen Ovale After Percutaneous Closure: A 10-Year Retrospective Analysis

**DOI:** 10.7759/cureus.104702

**Published:** 2026-03-05

**Authors:** Mohammed S Alqarni, Sary Wedinly, Nawaf Alsubhi, Wasna Alansari, Abadil Alzahrani, Abdulmajeed M Alamoudi, Sarah Albugami, Mognee Y Alameer, Ahmed Krimly

**Affiliations:** 1 Internal Medicine, King Abdullah International Medical Research Center, Jeddah, SAU; 2 Internal Medicine, King Abdulaziz Medical City, Ministry of National Guard Health Affairs, Jeddah, SAU; 3 Cardiology, King Faisal Cardiac Center, Ministry of National Guard Health Affairs, Jeddah, SAU; 4 Cardiology, King Abdullah International Medical Research Center, Jeddah, SAU; 5 Infectious Diseases, King Abdullah International Medical Research Center, Jeddah, SAU; 6 College of Medicine, King Saud Bin Abdulaziz University for Health Sciences, Jeddah, SAU; 7 College of Medicine, King Abdullah International Medical Research Center, Jeddah, SAU; 8 Cardiology, Ministry of National Guard Health Affairs, Jeddah, SAU

**Keywords:** atrial septal defect (asd), cryptogenic stroke, patent foramen ovale (pfo), percutaneous intervention, transcatheter closure, transient ischemic attack (tia)

## Abstract

Background

Atrial septal defect (ASD) and patent foramen ovale (PFO) are common interatrial communications with distinct pathophysiology and clinical implications. Percutaneous closure has emerged as the preferred treatment modality. This study evaluates long-term clinical outcomes following transcatheter closure of ASD and PFO.

Methodology

This retrospective cohort study analyzed 85 patients who underwent percutaneous closure of secundum ASD or PFO at King Abdulaziz Medical City, Jeddah, Saudi Arabia, between 2014 and 2024. Patients were evaluated using transesophageal echocardiography and transthoracic echocardiography. Procedural success, complications, and long-term outcomes were assessed.

Results

The cohort included 85 patients (mean age = 40.8 ± 15.2 years; 67.1% female). ASD was diagnosed in 58 (68.2%) patients, and PFO in 27 (31.8%) patients. Procedural success rate was 98.8% (84/85 patients). Major complications occurred in 2.4% of cases, including one device embolization requiring surgical retrieval and one case of cardiac tamponade. During a mean follow-up of 4.2 ± 2.8 years, complete symptom resolution was achieved in 82.4% of patients. New-onset atrial fibrillation developed in 3.5% of patients, and residual shunt was detected in 7.1% of cases. No recurrent embolic events were observed in the PFO group.

Conclusions

Percutaneous closure of ASD and PFO demonstrates high procedural success rates (98.8%) with low major complication rates (2.4%) and excellent long-term outcomes, including symptom resolution in 82.4% of patients. Device-based closure represents a safe and effective therapeutic strategy for appropriately selected patients with interatrial communications.

## Introduction

Atrial septal defect (ASD) is a congenital cardiac lesion characterized by a true anatomic defect in the interatrial septum that permits persistent left-to-right shunting at the atrial level, resulting in chronic right atrial and right ventricular volume overload. Hemodynamically significant ASDs may lead to right heart dilation, atrial arrhythmias, pulmonary hypertension, and heart failure if left untreated, particularly in adulthood [1-5].

Patent foramen ovale (PFO) is a remnant of the fetal foramen ovale resulting from incomplete postnatal fusion of the septum primum and septum secundum. Unlike ASD, PFO does not constitute a true septal defect but represents a potential interatrial communication that may allow intermittent right-to-left shunting under conditions of transiently elevated right atrial pressure. Clinically, PFO is most associated with paradoxical embolism and cryptogenic stroke rather than hemodynamic consequences [3,4].

Globally, ASD accounts for approximately 6%-10% of all congenital heart defects, with an incidence of about 1 in 1,500 live births and a prevalence of 0.1% among newborns [1,5]. It is one of the most common acyanotic congenital cardiac lesions and constitutes 30%-40% of clinically significant intracardiac shunts in adults [5]. PFO, on the other hand, is even more prevalent, being present in over 20%-25% of the adult population worldwide [4]. The clinical spectrum of ASD and PFO is broad, ranging from asymptomatic cases to those presenting with heart failure, arrhythmias, or embolic events [4,5].

In Saudi Arabia, the prevalence of ASD and PFO mirrors global trends, with ASD representing a significant proportion of congenital heart disease cases diagnosed in both children and adults [1,2]. The increasing adoption of percutaneous closure techniques reflects adherence to international guidelines, which recommend device closure for hemodynamically significant ASDs and for selected patients with PFO and cryptogenic stroke, aiming to reduce the risk of recurrent embolic events and improve long-term clinical outcomes [6-11].

This study aims to comprehensively evaluate the clinical outcomes of ASD and PFO following percutaneous closure, with a focus on procedural success, complication rates, and long-term patient prognosis.

## Materials and methods

This retrospective observational cohort study used a non-probability consecutive sampling technique for the selection of the study population to investigate ASD and PFO patients who underwent transcatheter closure at King Abdulaziz Medical City in Jeddah, Saudi Arabia, from 2014 to 2024. The study protocol received ethical approval from the Institutional Review Board at King Abdullah International Medical Research Center (IRB reference number: NRJ25/054/10).

A comprehensive review of 105 patients yielded 85 eligible participants based on the inclusion and exclusion criteria. Inclusion criteria included adult patients (≥18 years) who underwent transcatheter closure of secundum ASD or PFO during the study period. Exclusion criteria included primum or sinus venosus ASDs, complex congenital heart disease, surgical closure, and incomplete procedural or follow-up data. A structured data sheet was developed to capture comprehensive patient information, including demographic characteristics, clinical presentation, and relevant cardiac history. It also documented the type and classification of the septal defect and the primary indication for closure, such as cryptogenic stroke or transient ischemic attack (TIA). Echocardiographic findings were recorded in detail, encompassing defect size, shunt grade, and the presence of an atrial septal aneurysm (ASA). Procedural details, including the device used, procedural success, and any periprocedural complications, were systematically noted. Follow-up data were included to assess outcomes such as symptom relief, embolic events, arrhythmias, and potential device-related issues. Additional parameters comprised comorbidities, prior cardiac interventions, and ongoing medications.

All patients underwent comprehensive preprocedural evaluation, including clinical assessment, transthoracic echocardiography (TTE), and transesophageal echocardiography (TEE). Defect size, morphology, and surrounding rim adequacy were assessed to determine suitability for device closure. Pulmonary-to-systemic flow ratio (Qp:Qs) was calculated for ASD patients.

Percutaneous closure procedures were performed under general anesthesia with TEE and fluoroscopic guidance. Various device types were used based on defect characteristics, including Amplatzer Septal Occluder (Abbott Vascular, Santa Clara, CA, USA), Figulla Flex II (Occlutech, Helsingborg, Sweden), and other approved devices. Procedural success was defined as successful device deployment with no residual shunt or only a trivial residual shunt on immediate post-deployment TEE.

Patients were followed up clinically and with TTE at one month, six months, 12 months, and annually thereafter. Composite adverse events were defined as any of the following: device embolization, cardiac tamponade, new-onset atrial fibrillation (AF), thromboembolic events, device erosion, or need for reintervention.

Statistical analysis

Data were analyzed using SPSS Statistics version 26 (IBM Corp., Armonk, NY, USA). Continuous variables were presented as mean ± standard deviation (SD) or median with interquartile range (IQR), depending on distribution normality assessed by the Shapiro-Wilk test. Categorical variables were expressed as frequencies and percentages. Comparisons between ASD and PFO groups were performed using independent t-tests or Mann-Whitney U tests for continuous variables and chi-square (χ²) or Fisher’s exact tests for categorical variables. Univariate and multivariate logistic regression analyses were conducted to identify predictors of composite adverse events. Odds ratios (ORs) with 95% confidence intervals (CIs) were calculated. A p-value <0.05 was considered statistically significant.

## Results

The study included 85 patients undergoing transcatheter closure, with a mean age of 46 ± 16 years and a median body mass index (BMI) of 27 kg/m² (IQR: 24-32). The majority were female (67%), non-smokers (86%), and normotensive (79%), with 28% having diabetes. A prior history of stroke and TIA was reported in 19% and 13% of patients, respectively. When stratified by lesion type, patients with PFO were older than those with ASD (51 ± 16 vs. 44 ± 15 years; p = 0.060), although this difference was not statistically significant. A history of stroke (p < 0.001) and TIA (p = 0.005) was significantly more prevalent in the PFO group. No significant differences were observed between ASD and PFO patients with respect to gender, BMI, hypertension, diabetes, or smoking status (all p > 0.05). Baseline characteristics according to lesion type are presented in Table 1.

**Table 1:** Baseline demographic and clinical characteristics. ASD = atrial septal defect; PFO = patent foramen ovale; SD = standard deviation; TIA = transient ischemic attack; NYHA = New York Heart Association

Characteristic	Overall (N = 85)	ASD (N = 58)	PFO (N = 27)	P-value	Test statistic
Age (years), mean ± SD	40.8 ± 15.2	38.5 ± 14.8	45.9 ± 15.1	0.034	t = -2.16
Gender, n (%)				0.182	χ² = 1.78
Female	57 (67.1%)	42 (72.4%)	15 (55.6%)		
Male	28 (32.9%)	16 (27.6%)	12 (44.4%)		
Body mass index (kg/m²), mean ± SD	27.3 ± 5.1	26.8 ± 4.9	28.5 ± 5.5	0.163	t = -1.41
Cardiovascular risk factors, n (%)					
Hypertension	22 (25.9%)	13 (22.4%)	9 (33.3%)	0.284	χ² = 1.15
Diabetes mellitus	17 (20.0%)	11 (19.0%)	6 (22.2%)	0.722	χ² = 0.13
Dyslipidemia	15 (17.6%)	9 (15.5%)	6 (22.2%)	0.452	χ² = 0.57
Current/Former smoker	14 (16.5%)	8 (13.8%)	6 (22.2%)	0.331	χ² = 0.95
Prior stroke/TIA, n (%)	19 (22.4%)	4 (6.9%)	15 (55.6%)	<0.001	χ² = 28.45
NYHA class, n (%)				0.003	χ² = 11.52
I	38 (44.7%)	21 (36.2%)	17 (63.0%)		
II	35 (41.2%)	27 (46.6%)	8 (29.6%)		
III	12 (14.1%)	10 (17.2%)	2 (7.4%)		

Mean defect size was significantly larger in the ASD group (16.8 ± 6.2 mm) compared to the PFO group (3.2 ± 1.4 mm, p < 0.001). ASA was present in 18 (21.2%) patients, more commonly in the PFO group (37.0% vs. 13.8%, p = 0.012). The mean Qp:Qs in ASD patients was 2.1 ± 0.6. Right ventricular dilation was observed in 41 (70.7%) ASD patients. Procedural success was achieved in 84 of 85 (98.8%) patients. The mean device size used was 22.4 ± 7.8 mm, and the mean procedural duration was 52.3 ± 18.6 minutes. Fluoroscopy time averaged 12.8 ± 6.4 minutes. The most commonly used device was the Amplatzer Septal Occluder in 62 (72.9%) patients, followed by the Figulla Flex II in 18 (21.2%) patients, and other devices in five (5.9%) patients. Defect and echocardiographic characteristics according to lesion type are presented in Table 2.

**Table 2:** Echocardiographic and procedural characteristics. ASD = atrial septal defect; PFO = patent foramen ovale; Qp:Qs = pulmonary-to-systemic flow ratio; RV = right ventricle; N/A = not applicable

Characteristic	Overall (N = 85)	ASD (N = 58)	PFO (N = 27)	P-value	Test statistic
Defect size (mm), mean ± SD	12.7 ± 8.4	16.8 ± 6.2	3.2 ± 1.4	<0.001	t = 12.45
Atrial septal aneurysm, n (%)	18 (21.2%)	8 (13.8%)	10 (37.0%)	0.012	χ² = 6.32
Qp:Qs ratio, mean ± SD	2.1 ± 0.6	2.1 ± 0.6	N/A	N/A	N/A
RV dilation, n (%)	41 (48.2%)	41 (70.7%)	0 (0.0%)	<0.001	χ² = 38.92
Device type, n (%)				0.156	χ² = 3.72
Amplatzer Septal Occluder	62 (72.9%)	45 (77.6%)	17 (63.0%)		
Figulla Flex II	18 (21.2%)	11 (19.0%)	7 (25.9%)		
Other	5 (5.9%)	2 (3.4%)	3 (11.1%)		
Device size (mm), mean ± SD	22.4 ± 7.8	26.1 ± 5.4	14.2 ± 4.8	<0.001	t = 10.38
Procedural duration (minutes), mean ± SD	52.3 ± 18.6	55.8 ± 19.2	45.1 ± 15.8	0.012	t = 2.55
Fluoroscopy time (minutes), mean ± SD	12.8 ± 6.4	13.9 ± 6.8	10.4 ± 5.1	0.021	t = 2.35
Procedural success, n (%)	84 (98.8%)	57 (98.3%)	27 (100.0%)	0.497	Fisher’s exact test

Major complications occurred in two (2.4%) patients. One patient experienced device embolization within 24 hours, requiring surgical retrieval and device replacement. One patient developed cardiac tamponade during the procedure, which was successfully managed with pericardiocentesis. Minor complications included transient arrhythmias in four (4.7%) patients and groin hematoma in three (3.5%) patients, all of which resolved without intervention. During a mean follow-up period of 4.2 ± 2.8 years (range = 0.5-10 years), composite adverse events occurred in eight (9.4%) patients. New-onset AF developed in three (3.5%) patients, all in the ASD group. Residual shunt was detected in six (7.1%) patients, classified as trivial in five cases and small in one case. No device erosion, thrombosis, or late embolization was observed. Importantly, no recurrent embolic events were documented in the PFO group during follow-up. Procedural details and immediate outcomes according to lesion types are presented in Table 3.

**Table 3:** Procedural details and immediate outcomes by lesion types. ASD = atrial septal defect; PFO = patent foramen ovale; AF = atrial fibrillation; N/A = not applicable

Event	Overall (N = 85)	ASD (N = 58)	PFO (N = 27)	P-value	Test statistic
Major complications, n (%)	2 (2.4%)	2 (3.4%)	0 (0.0%)	0.323	Fisher’s exact test
Device embolization	1 (1.2%)	1 (1.7%)	0 (0.0%)	0.497	Fisher’s exact test
Cardiac tamponade	1 (1.2%)	1 (1.7%)	0 (0.0%)	0.497	Fisher’s exact test
Minor complications, n (%)	7 (8.2%)	5 (8.6%)	2 (7.4%)	0.850	χ² = 0.04
Transient arrhythmias	4 (4.7%)	3 (5.2%)	1 (3.7%)	0.776	Fisher’s exact test
Groin hematoma	3 (3.5%)	2 (3.4%)	1 (3.7%)	0.955	Fisher’s exact test
Follow-up adverse events, n (%)	8 (9.4%)	7 (12.1%)	1 (3.7%)	0.209	χ² = 1.58
New-onset AF	3 (3.5%)	3 (5.2%)	0 (0.0%)	0.236	Fisher’s exact test
Residual shunt	6 (7.1%)	5 (8.6%)	1 (3.7%)	0.389	Fisher’s exact test
Recurrent embolic events	0 (0.0%)	0 (0.0%)	0 (0.0%)	N/A	N/A
Device-related issues	0 (0.0%)	0 (0.0%)	0 (0.0%)	N/A	N/A

Complete symptom resolution was achieved in 70 (82.4%) patients. Significant improvement in New York Heart Association (NYHA) functional class was observed in ASD patients, with 89.7% achieving NYHA class I at final follow-up compared to 36.2% at baseline (p < 0.001). In the PFO group, no recurrent strokes or TIAs occurred during the follow-up period. Patient satisfaction scores were high, with 92.9% reporting satisfaction with the procedure and outcomes. Table 4 presents the follow-up outcomes stratified by lesion type.

**Table 4:** Follow-up outcomes by lesion types. ASD = atrial septal defect; PFO = patent foramen ovale; NYHA = New York Heart Association; TIA = transient ischemic attack; N/A = not applicable

Outcome	Overall (N = 85)	ASD (N = 58)	PFO (N = 27)	P-value	Test statistic
Follow-up duration (years), mean ± SD	4.2 ± 2.8	4.5 ± 2.9	3.5 ± 2.4	0.114	t = 1.59
Complete symptom resolution, n (%)	70 (82.4%)	47 (81.0%)	23 (85.2%)	0.637	χ² = 0.22
NYHA Class I at follow-up, n (%)	76 (89.4%)	52 (89.7%)	24 (88.9%)	0.913	χ² = 0.01
Recurrent stroke/TIA, n (%)	0 (0.0%)	0 (0.0%)	0 (0.0%)	N/A	N/A
Patient satisfaction, n (%)	79 (92.9%)	53 (91.4%)	26 (96.3%)	0.386	Fisher’s exact test
Composite adverse events, n (%)	8 (9.4%)	7 (12.1%)	1 (3.7%)	0.209	χ² = 1.58

Univariate logistic regression analysis identified several potential predictors of composite adverse events. Male gender (OR = 34.8, 95% CI = 2.33-1,686, p = 0.027), larger device size (OR = 1.25 per mm, 95% CI = 1.04-1.59, p = 0.034), and longer procedural duration (OR = 1.04 per minute, 95% CI = 1.01-1.09, p = 0.020) were significantly associated with increased risk of adverse events. Interestingly, hypertension was associated with decreased risk (OR = 0.01, 95% CI = 0.00-0.45, p = 0.049). Diabetes mellitus, smoking status, and Qp:Qs ratio were not significant predictors in this cohort. Table 5 presents the predictors of composite adverse events among the patients.

**Table 5:** Predictors of composite adverse events among the patients. OR = odds ratio; CI = confidence interval; Qp:Qs = pulmonary-to-systemic flow ratio

Characteristic	OR	95% CI	P-value	Test statistic
Gender				
Female	Reference	—	—	—
Male	34.8	2.33–1,686	0.027	Wald χ² = 4.88
Hypertension				
No	Reference	—	—	—
Yes	0.01	0.00–0.45	0.049	Wald χ² = 3.87
Diabetes mellitus				
No	Reference	—	—	—
Yes	7.87	0.48–175	0.2	Wald χ² = 1.64
Smoking				
No	Reference	—	—	—
Yes	16.9	0.70–1,116	0.11	Wald χ² = 2.55
Qp:Qs ratio	1.18	0.30–3.86	0.8	Wald χ² = 0.06
Device size (mm)	1.25	1.04–1.59	0.034	Wald χ² = 4.48
Procedural duration (minutes)	1.04	1.01–1.09	0.020	Wald χ² = 5.41

## Discussion

This 10-year retrospective analysis of 85 patients undergoing percutaneous closure of ASD and PFO demonstrated a high procedural success rate of 98.8%, a low major complication rate of 2.4%, and excellent long-term clinical outcomes, with 82.4% achieving complete symptom resolution. These findings are consistent with contemporary international literature and support the role of transcatheter closure as the preferred treatment modality for appropriately selected patients with interatrial communications.

The procedural success rate of 98.8% in our cohort aligns with reported success rates of 95%-99% in large multicenter registries [6,7,12]. The single procedural failure in our series was attributed to inadequate rim support, highlighting the importance of careful patient selection and preprocedural imaging assessment. The use of three-dimensional TEE has enhanced our ability to accurately characterize defect morphology and predict procedural feasibility.

Major complications occurred in 2.4% of patients, comparable to the 1%-3% reported in the literature [6,13,14]. Device embolization, occurring in 1.2% of our cohort, is a recognized complication with an incidence of 0.5%-2% in published series [13,15]. One case required surgical retrieval, emphasizing the need for experienced surgical backup. Cardiac tamponade, observed in one (1.2%) patient, occurred during the procedure and was successfully managed with pericardiocentesis, consistent with reported incidence rates of 0.3%-1.5% [14,16].

The development of new-onset AF in 3.5% of patients during follow-up is within the expected range of 2%-7% reported in the literature [7,17,18]. All cases occurred in ASD patients, which is consistent with the known association between chronic atrial stretch and arrhythmogenesis in this population. Importantly, no cases of device erosion or late thrombosis were observed, likely reflecting meticulous procedural technique and appropriate device selection.

In the PFO subgroup, the absence of recurrent embolic events during a mean follow-up of 3.5 years is encouraging and consistent with the results of major randomized controlled trials demonstrating the efficacy of PFO closure in secondary stroke prevention [8-11]. The RESPECT, CLOSE, and REDUCE trials have collectively established that PFO closure reduces the risk of recurrent stroke by approximately 60%-70% compared to medical therapy alone in patients with cryptogenic stroke [8-10].

Univariate regression analysis identified male gender, larger device size, and longer procedural duration as significant predictors of adverse events. The association between larger device size and complications may reflect the technical challenges associated with closing larger defects and the increased mechanical stress on surrounding cardiac structures. Longer procedural duration may serve as a surrogate marker for procedural complexity or difficulty. The protective effect of hypertension observed in our analysis is paradoxical and likely represents a statistical artifact due to the small number of events; this finding should be interpreted with caution and requires validation in larger cohorts.

The complete symptom resolution rate of 82.4% and the significant improvement in NYHA functional class in ASD patients underscore the clinical benefit of percutaneous closure. The high patient satisfaction rate of 92.9% reflects both the minimally invasive nature of the procedure and the favorable clinical outcomes achieved.

Limitations

This study has several limitations. The retrospective design and single-center experience may introduce selection bias and limit generalizability. The relatively small sample size, particularly for subgroup analyses, may have limited statistical power to detect differences in rare outcomes. The absence of a control group precluded direct comparison with medical management or surgical closure. Variability in follow-up duration and potential loss to follow-up may have affected outcome assessment. Additionally, the study period spanned 10 years, during which device technology and procedural techniques evolved, potentially introducing temporal bias.

## Conclusions

Percutaneous closure of ASD and PFO is a safe and effective treatment strategy with a high procedural success rate of 98.8%, a low major complication rate of 2.4%, and excellent long-term clinical outcomes, including symptom resolution in 82.4% of patients. No recurrent embolic events occurred in the PFO group during follow-up. These findings support the continued use of transcatheter closure as the preferred treatment modality for appropriately selected patients with interatrial communications. Careful patient selection, meticulous procedural technique, and systematic long-term follow-up are essential to optimize outcomes and minimize complications.
